# Flow Cytometry of Microencapsulated Colonies for Genetics Analysis of Filamentous Fungi

**DOI:** 10.1534/g3.114.014357

**Published:** 2014-09-19

**Authors:** Lidia Delgado-Ramos, Ana T. Marcos, María S. Ramos-Guelfo, Leyre Sánchez-Barrionuevo, Francis Smet, Sebastián Chávez, David Cánovas

**Affiliations:** * Departamento de Genética, Hospital Universitario Virgen del Rocío-CSIC-Universidad de Sevilla, Seville, Spain; † Instituto de Biomedicina de Sevilla (IBiS), Hospital Universitario Virgen del Rocío-CSIC-Universidad de Sevilla, Seville, Spain; ‡ Union Biometrica, Geel, Belgium

**Keywords:** filamentous fungi, *Aspergillus*, flow cytometry, microencapsulation, genetic analysis

## Abstract

The analysis of filamentous fungi by flow cytometry has been impossible to date due to their filamentous nature and size. In this work, we have developed a method that combines single-spore microencapsulation and large-particle flow cytometry as a powerful alternative for the genetic analysis of filamentous fungi. Individual spores were embedded in monodisperse alginate microparticles and incubated in the appropriate conditions. Growth could be monitored by light or fluorescent microscopy and Complex Object Parametric Analyzer and Sorter large-particle flow cytometry. Microencapsulated *Trichoderma* and *Aspergillus* spores could germinate and grow inside the alginate capsules. Growth tests revealed that auxotrophic mutants required the appropriate nutrients and that pyrithiamine and glufosinate halted fungal growth of sensitive but not resistant strains. We used an *Aspergillus nidulans*, thermosensitive mutant in the cell-cycle regulator gene *nimX^CDK1^* as proof-of-concept to the detection and identification of genetic phenotypes. Sorting of the microparticles containing the clonal fungal mycelia proved the power of this method to perform positive and/or negative selection during genetic screenings.

Filamentous fungi constitute a very diverse biological group, with a great impact on human life (May and Adams 1997). Production of antibiotics, food additives, or recombinant proteins at industrial scale are good examples of the biotechnological importance of filamentous fungi (Brakhage 2013; Knuf and Nielsen 2012; Ozcengiz and Demain 2013). Several filamentous fungal species are pathogens of plants and metazoans, including humans (Sexton and Howlett 2006). In basic research, filamentous fungal models have contributed to establish the basis of key biological aspects such as genetic information flow and metabolism (Beadle and Tatum 1941), DNA recombination (Holliday 1964), or gene silencing (Cogoni and Macino 1999). In all these fields of fungal research, genetic analysis is one of the most productive tools. Classic, forward, and reverse genetic tools are readily available for fungal models such as *Aspergillus* and *Neurospora*, and genetic approaches still remain a fundamental source for the discovery of novel biological functions. However, genetic screenings with filamentous fungi usually involve laborious, tedious, and/or time-consuming procedures (for examples, see Harris *et al.* 1994; Seiler and Plamann 2003), due to the formation of heterokaryons in organisms that undergo cell fusion and the growth mode, forming hyphae with multinuclear cellular compartments and tangled webs of mycelia instead of undergoing budding or cell fission to form two distinct uninucleated daughter cells from one mother. A good example of these limitations is cell-cycle research, a field in which the *Aspergillus* model emerged as early as the yeast ones (Hartwell *et al.* 1970; Morris 1975; Nurse 1975). However, due to the simplicity of yeast procedures, research with these single-cell organisms advanced more rapidly.

These drawbacks of filamentous fungal research can be solved, at least partially, with the use of high-throughput methods like, for instance, the combination of flow cytometry and sorting. The problem is that flow cytometry machines are not suitable for filamentous organisms. Recently, large-particle flow cytometry has been applied to fungal pellets (de Bekker *et al.* 2011), but fungal pellets usually contain a mixture of several fungal individuals, and, as such, they can be considered fungal populations rather than clonal fungal entities. In this work, we overcome all these issues with a novel method for generating isolated fungal microcolonies after the germination of microencapsulated spores, which makes possible their analysis by flow cytometry and sorting. This method allows for positive and negative selection during genetic screenings.

## Materials and Methods

### Strains, media, and culture conditions

The fungal species used in this work are *T. reesei* strain QM9414 and *A. nidulans*. The *A. nidulans* strains used in this study are listed in Table 1. Strains were grown in complete (CMA) or minimal (MMA) media containing the appropriate supplements (Cove 1966). Experiments performed with thermosensitive strains were carried out at 30 or 42° as indicated. Glucose was used as sole carbon source, and ammonium tartrate was used as sole nitrogen source. Strains were obtained by following standard procedures (Pontecorvo *et al.* 1953). Pyrithiamine was obtained from Sigma and used at 0.1 µg/mL. Glufosinate was prepared from Basta (Bayer CropScience) and used as previously described (Nayak *et al.* 2006).

**Table 1:** *A. nidulans* strains used in this study

Strain	Genotype	Source
FGSC4	Wild-type strain, *veA*^+^	FGSC
DKA63	*biA1*	
DKA4	*pyroA4 pyrG89 wA2*	M. Peñalva
DKA95(AGB288)	*∆cryA*::*ptrA veA*^+^	G. Braus
DKA200 (FGSC A1096)	*pabaA1*	FGSC
DKA13(MH11057)	*pabaA1 niiA4 ∆nkuA*::*bar yA1*	M. Hynes
HA344	*H1-chRFP*::*Afpyro pyrG89 (pyroA4*; *argB2?) ∆nkuA*::*argB*	S. Osmani
DKA180	*nimX2^F233L^*, *yA2*, *nicB8*, *pyroA4*; *HH1-GFP-Afpyro*^+^; *∆nkuA (?)*	This study
Strains used only for crossings		
FGSC A1126	*nimX2^F233L^ nicB8 pyroA4 yA2*	Osmani *et al.* (1994), obtained from FGSC
LO 1945	*pyrG89 riboB2 pyroA4 nkuA∆ HH1-GFP-Afpyro*^+^	B. Oakley

All strains are *veA1*, unless stated. FGSC, Fungal Genetics Stock Center.

### Microencapsulation of fungal spores

**Figure 1:**
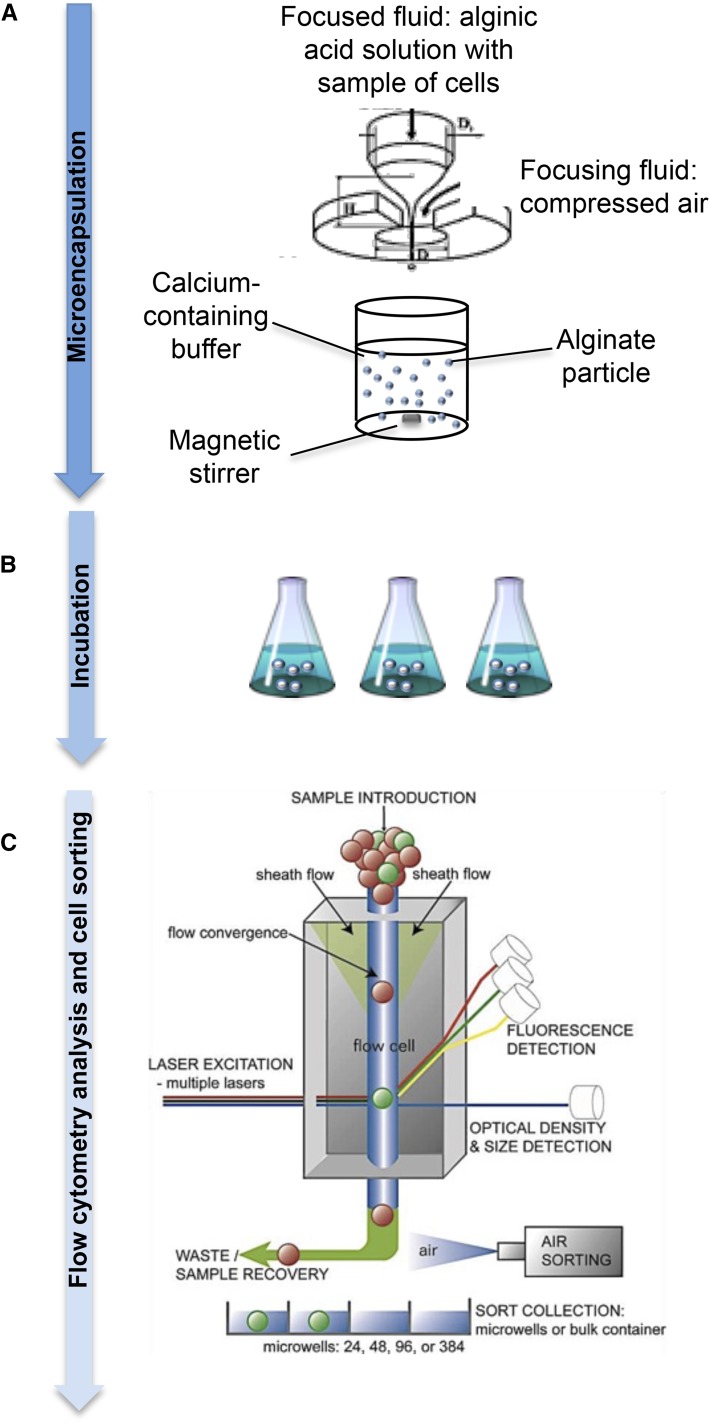
Summary of the method: microencapsulation, incubation, and sorting processes. (A) A schematic representation of the flow-focusing technology is shown. The sample is injected with a syringe pump through a capillary feed tube inside a chamber and pressurized by a continuous air supply in a Cellena microencapsulator. The stationary jet breaks up by capillary instability into homogeneous droplets, which will gel in a continuously stirred calcium chloride solution at room temperature. Adapted from Martin-Banderas *et al.* (2005). (B) Spherical size-monodisperse alginate microcapsules containing the fungal spores are incubated in different media. (C) Flow cytometry and cell-sorting analysis: microcapsules containing single cell were analyzed by flow cytometry using a COPAS flow cytometer. COPAS instruments allow one to automate the process of sorting large particles (20−1500 µm) in a continuously flowing stream at a rate of 10−50 objects/sec. Via the use of object size (time of flight), optical density (extinction), and/or intensity of fluorescent markers as sorting criteria, selected objects in a predetermined range can be safely dispensed in multiwell plates for further analysis.

**Figure 2:**
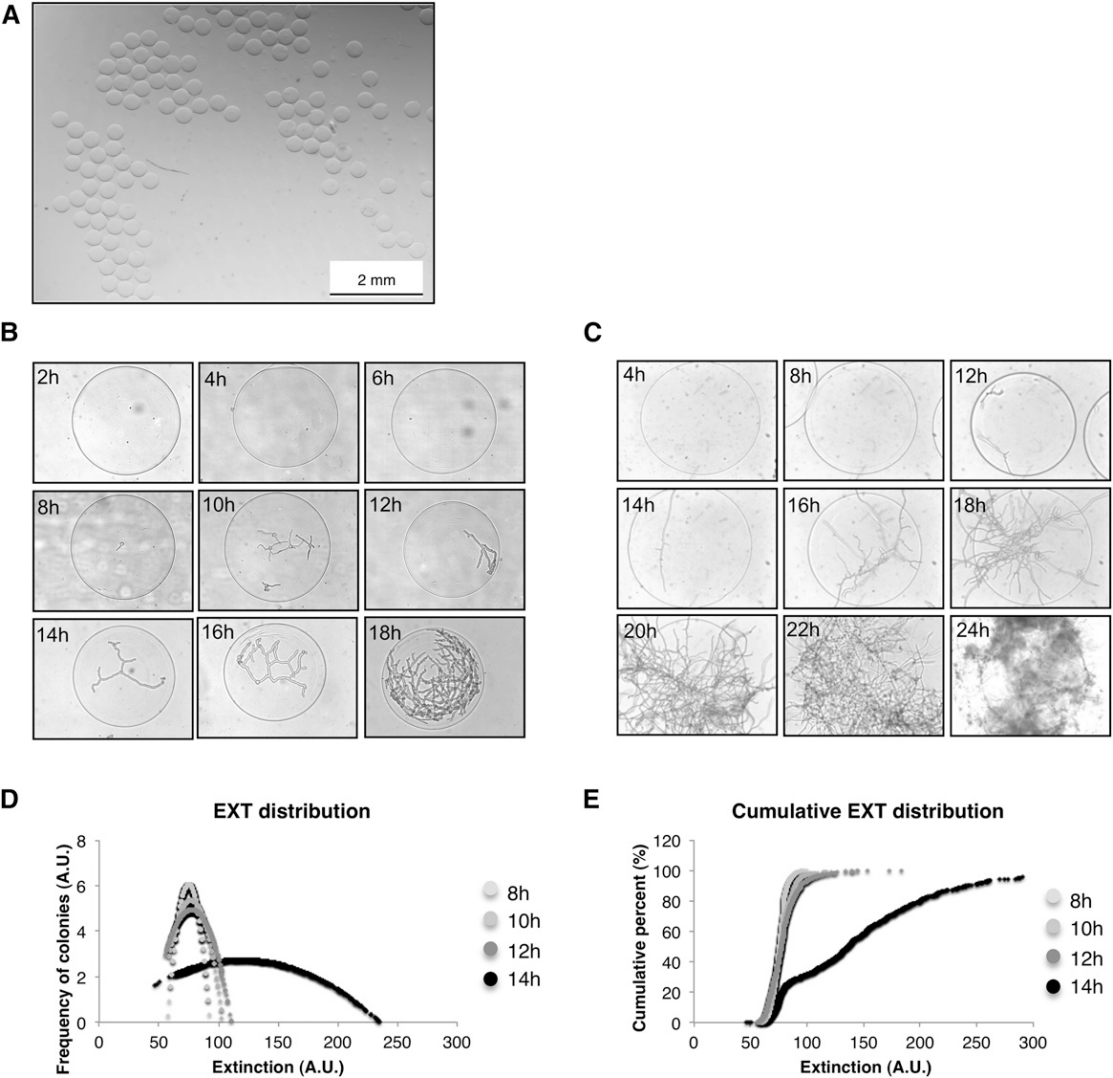
Monitoring the proliferation of *T. reesei* and *A. nidulans* spores by light microscopy and flow cytometry. (A) Spherical size-monodispersed alginate microcapsules fabricated in the microencapsulator. (B−C) Pictures of encapsulated spores during germination. In this test the spores were encapsulated in 400-μm, 3% alginate capsules. After encapsulation, the beads were incubated in shaking flasks and samples were recovered after different time of incubation. (B) *Trichoderma reesei*. (C) *A. nidulans*. (D−E) Flow cytometry analysis of encapsulated spores *T. reesei*. Aliquots were analyzed by COPAS SELECT flow cytometry allowing the measurement of different optical parameters: size (time of flight), optical density (EXT), green self-fluorescence and red self-fluorescence signals. The germination of spores is associated with an increase in density. These measurements are represented in the graphs showing the increase of EXT over time (left) and the EXT distribution within the bead (right). A.U., arbitrary unit; EXT, extinction.

Fungal spores were microencapsulated in calcium alginate beads (400 µm) in a Cellena microencapsulator device (Ingeniatrics) following the manufacturer’s instructions as previously described (Gomez-Herreros *et al.* 2012) (Figure 1A). Microencapsulation conditions were adjusted to obtain single-spore capsules, which ensures that all the mycelial cells growing inside the microcapsules derived from one single spore and consequently, they are clonal. This procedure gave microcapsules with a regular spherical shape and homogenous size, which contained one spore inside the beads (Figure 2A). Spore suspensions were adjusted at 0,160 units at O.D. 600 nm, and 10 µL of each suspension was mixed with 290 µL of water and 2.7 mL of alginate 3%. The sample was then injected with a syringe pump through a capillary feed tube inside a chamber and pressurized by a continuous air supply (Figure 1A). The stationary jet broke up by capillary instability into homogeneous droplets, which gel in a continuously stirred 3% calcium chloride solution at room temperature. The capsules were stored in the same solution at 4° with agitation for at least 1 hr or until they were used, then they were filtered and washed with distilled water to remove excess of calcium chloride. Then, microcapsules were inoculated with a sterile spoon into liquid media and incubated under the appropriate conditions.

### Microscopy

Capsules containing fungal strains were grown in CMA or MMA liquid medium with appropriate supplements at the indicated temperature. Capsules were examined using light or fluorescent microscopy and visualized on a Leica DMR fluorescent microscope with the appropriate filter sets and equipped with a Leica DC350F camera, or Leica DM1000 light microscope equipped with a Leica EC3 camera. Shape and homogeneity of the capsules and results after sorting were analyzed in a Leica M125 stereo microscope equipped with a Leica DFC450C camera.

### Complex Object Parametric Analyzer and Sorter (COPAS) large-particle flow cytometry

Microencapsulated fungal spores were grown in the indicated media. After we confirmed hyphal growth by light microscopy, the population of microencapsulated colonies was analyzed in a Complex Object Parametric Analyzer and Sorter (COPAS) SELECT flow cytometer (Union Biometrica) (Figure 1C). Relative microcolony size was monitored by measuring the axial length of the object (time of flight), the optical density of the detected object (optical extinction) and fluorescence under the following photomultiplier tube settings: green (1100), yellow (600), and red (1100). The time of flight minimum was fixed at 150, and the extinction signal was 3.1. Sheath fluid pressure was adjusted to 4.40–5.20, whereas sample fluid pressure was set to maintain a frequency of 15–25 events per second.

For the sorting assay, the optimal parameters were fixed to perform the selection of the population of interest as required. After sorting, the particles were collected in Petri dishes and analyzed by visualization under stereo and fluorescent microscopy. To recover the encapsulated strains, particles were plated on complete solid media and incubated at 30° (permissive temperature) for 3−4 d.

## Results and Discussion

### Generation of fungal microcolonies by spore microencapsulation

Fungal spores were microencapsulated in calcium alginate beads with conditions adjusted to obtain single-spore capsules. This procedure gave microcapsules with a regular spherical shape and a homogenous size close to 400 µm (Figure 1A and Figure 2A). Microcapsules were first inoculated into liquid media to test the capacity of two selected fungal species, *Trichoderma reesei* and *Aspergillus nidulans*, to grow inside the alginate microcapsules (Figure 1B and Figure 2, B and C). Samples were taken at regular intervals, and fungal growth was monitored by light microscopy. Alginate beads are transparent, which allowed for fungal visualization under the light microscope. Germination of spores inside the microcapsules could be observed 8 hr after inoculation. Fungal growth proceeded normally inside the alginate microcapsules by apical extension and branching. Proliferation inside the capsules could be measured by flow cytometry using the COPAS technology (Figure 1C). Particles exhibiting higher optical density increased with the time of incubation (Figure 2, D and E). Cumulative distribution of optical density indicated that after 14 hr mycelia accumulation was heterogeneous, suggesting that some microcolonies were growing faster than other, as was also observed with microscopy (Figure 2, B−E). The parallelism between microscopic observation and flow cytometry measurement indicates that the combination of spore microencapsulation and COPAS technology is a valid high-throughput method for the analysis of filamentous fungal growth.

### Detection of commonly used phenotypes of microencapsulated fungi

**Figure 3:**
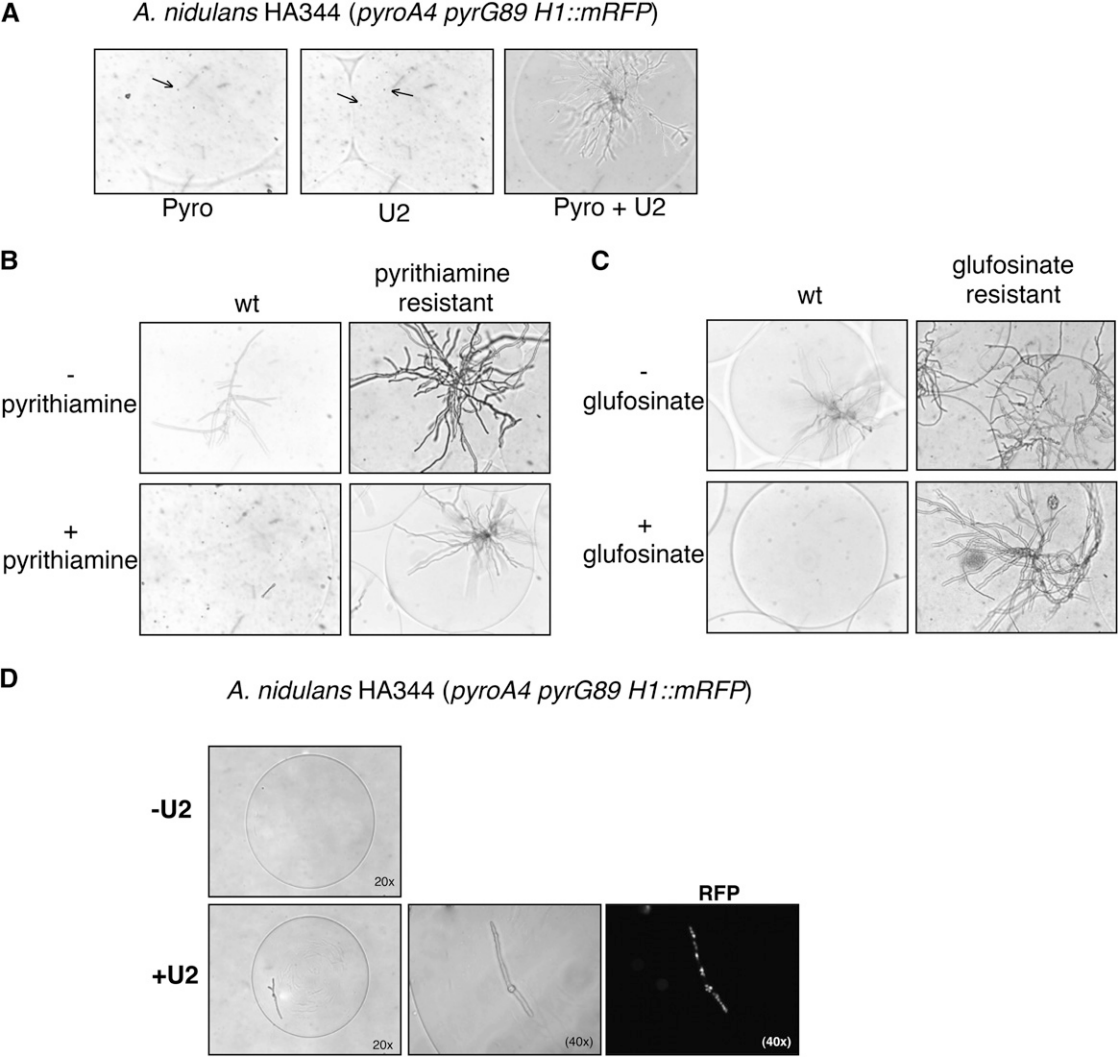
Fungal phenotypes can be detected in microcapsules. Encapsulation allows one to screen for growth/no growth and/or fluorescence. In (A) the auxotroph mutant HA344: H1::RFP *pyrG89 pyroA4* is not able to grow in the absence of pyridoxine or U2 (uracil + uridine). This screening can be extended to search for resistance to different compounds. For each case, we used a fungicide-resistant strain obtained by transformation of a resistance cassette in the genome and the corresponding isogenic strain. Capsules containing spores were incubated in minimal media in the presence or absence of pyrithiamine (B) and glufosinate (C) for 25−30 hr. (D) When nutritional requirements were added to the medium, the spores started to germinate and grow by apical elongation as shown by fluorescence microscopy.

To test the reliability of this method and its utility for genetic analysis, different assays were performed. First, we tested whether microcapsules were suitable systems for detecting common fungal phenotypes. Proliferation of an auxotrophic *A. nidulans* mutant (*pyroA4 pyrG89*) was only detected in the microcapsules when the corresponding supplements were present in the medium (Figure 3A). In agreement with previous observations (Osmani *et al.* 2006), the auxotrophic spores could germinate in the absence of U2, but growth soon stopped, resulting in very short hyphae. Similarly, strains resistant to two different fungicides (pyrithiamine and glufosinate) were capable of colonizing microcapsules in the presence of the drugs, whereas isogenic non-resistant strains were unable, as expected (Figure 3, B and C). Finally, strains expressing fluorescent-tagged proteins were visualized when growing inside the microcapsules (Figure 3D).

### Flow cytometry analysis of encapsulated fungal microcolonies

**Figure 4:**
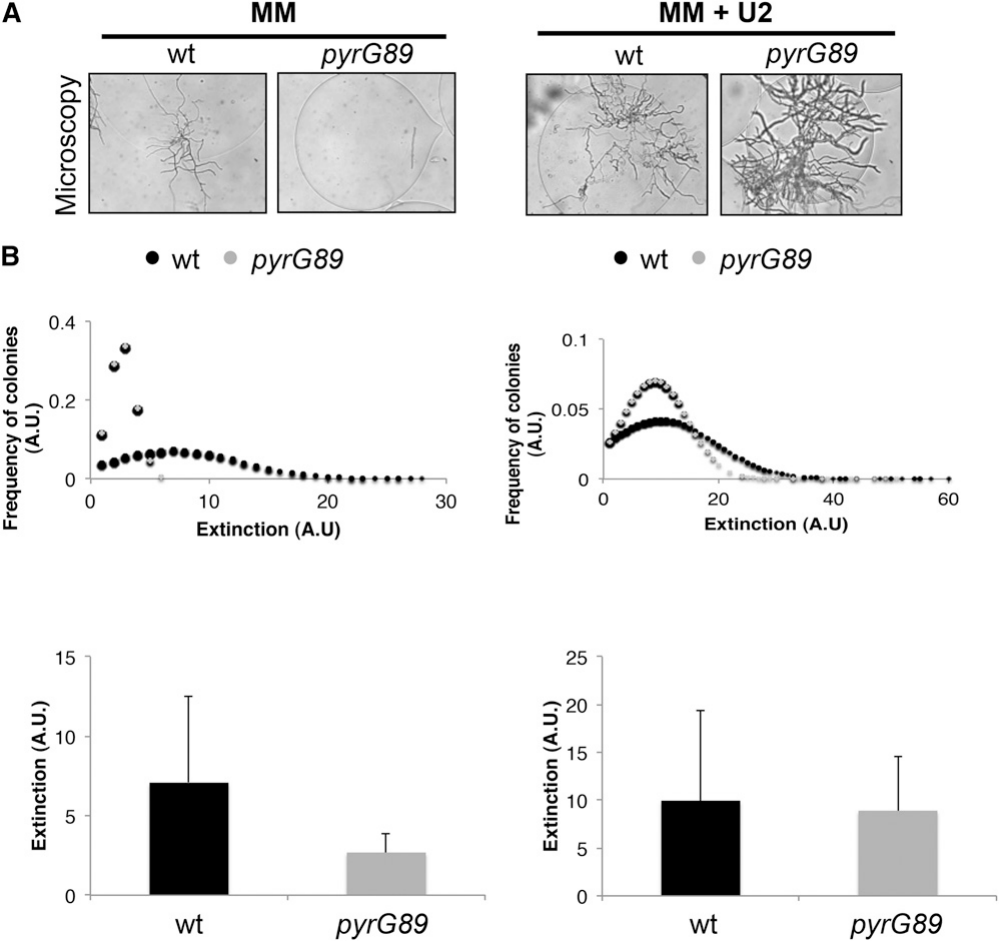
Screening for fungal growth or fluorescence. Pictures of encapsulated *A. nidulans* spores after growing in different media and flow cytometry analysis of the particles. A *pyrG89* strain (auxotrophic for uracil and uridine) carrying the histone H1-RFP fusion and a nonfluorescent wild-type strain were grown in minimal media in the absence or in the presence of U2 for 20 hr. Fungal growth in the microcapsules was visualized by light microscopy (A) and quantified by COPAS flow cytometry showing the extinction levels (B).

COPAS flow cytometry allowed the analysis of these phenotypes across the microencapsulated population in a quantitative manner. Cytometric analysis detected a population of wild-type microcolonies with increased optical density that was missing in the microcapsules containing auxotrophic *pyrG89* spores (Figure 4). In contrast, a prominent population of microcapsules with increased optical density was detected when the auxotrophic mutant was incubated in the presence of uracil and uridine (Figure 4). In the presence of U2, we were able to detect signal in the red channel in the case of the *pyrG89* strain arising from all the H1-RFP−tagged nuclei. Despite of the wild-type growing, signal in the red channel was not observed (data not shown).

### Genetic screening of microencapsulated fungi by large particle flow cytometry

**Figure 5:**
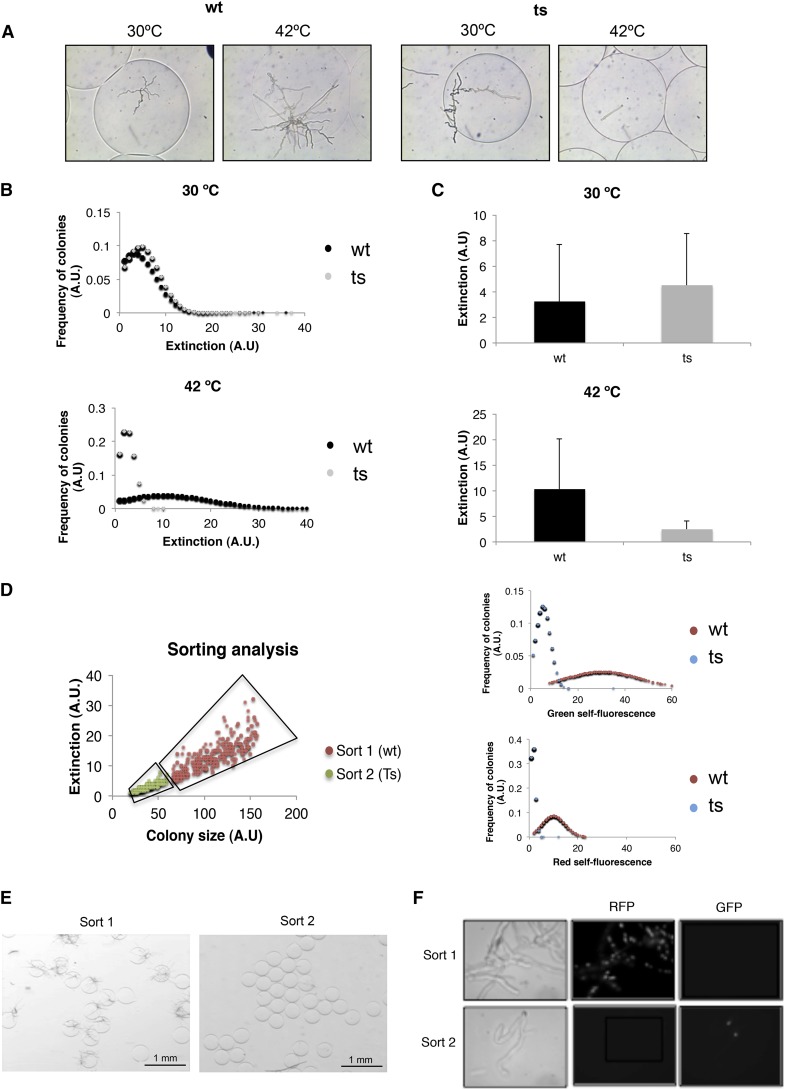
Screening for conditional mutants using thermosensitive (ts) alleles. (A) Analysis of ts mutants by microencapsulation. In the left box is shown the growth of single spores of the wild-type (wt) *A. nidulans* at permissive and restrictive temperature, and in the right the ts strain is shown. (B-C) Flow cytometry analysis of encapsulated wt and ts *A. nidulans* spores after growing at different temperatures showing the measurement of optical density (EXT) at both temperatures. (D) Sorting analysis of wt and ts mutant. The spots depict the population of interest in both cases, showing green and red self-fluorescence (diagrams on the right). (E) Pictures of the cell-sorting results of both strains grown at restrictive temperatures: sort 1 (wt strain) and sort 2 (ts atrain) under optical microscope. (F) Confirmation of the identity of the strains after sorting was performed taking advantage of the H1::chRFP (wt) and HH1::GFP (ts mutant) fusion proteins under fluorescence microscope. EXT, extinction.

Thermosensitive mutants (ts) are very useful tools for studying the function of essential genes (Govindaraghavan *et al.* 2014). However, isolation of such mutants requires extremely laborious replica plating of large number of mutants (Harris *et al.* 1994). We figured that our procedure could be used for the isolation of ts mutants in essential genes. As a proof-of-concept we used a ts mutation in the *nimX* gene, which encodes the cyclin-dependent protein kinase involved in cell-cycle control CDK1/CDC2 (Osmani *et al.* 1994). Wild-type and ts mutant strains were capable of proliferate in the microcapsules at the permissive temperature, as visualized by light microscopy and detected by COPAS flow cytometry. In contrast, only the wild-type microcolonies were detected at the restricted temperature. As expected, the wild type grew faster at 42 than at 30° (Figure 5, A-C). In a parallel experiment, these two types of microcapsules, containing spores of either the wild type or the mutant, were mixed together and incubated at the restrictive temperature for 18 hr. Two populations were identified when the mixture of microcapsules was analyzed by COPAS flow cytometry, based on their optical parameters. One was distinguished by its high optical density and time of flight values; the other one was low values for both parameters (Figure 5D). Both populations were sorted independently. To check the quality of the sorting, microcapsules were visualized under a stereo microscope, revealing that most of the microcapsules of one of the populations carried grown mycelia opposite to the other population, in which microcapsules did not carry grown mycelia (Figure 5E). Both strains could be easily distinguished by their fluorescence. The wild-type strain carried a H1::RFP fusion, whereas the ts mutant carried a H1::green fluorescent protein fusion (Figure 5D). Visualization of the sorted microcapsules under the fluorescent microscope confirmed that the population of grown mycelia contained the wild-type strain (red fluorescent), whereas the nongrown population contained the ts short hyphae of the mutant strain (green fluorescent) (Figure 5F). These microcapsules could then be plated on regular Petri dishes and incubated on complete media at the permissive temperature to recover the desired strains, either wild type or mutant (data not shown).

This method allows for the analysis of clonal fungal colonies grown in liquid media by flow cytometry. The power of this method relies on the capacity to do genetic screenings and make both positive and negative selection, for example, allowing to sort strains carrying ts alleles of essential genes, which could be later recovered by growing at the permissive temperature. Performing this method skips the laborious and time-consuming requirement of replica plating. In addition, this method can be extended to screenings for regulators by employing GFP genes under the control of a promoter of interest. It makes this method powerful and versatile.
